# Deep Learning Combined with Radiologist's Intervention Achieves Accurate Segmentation of Hepatocellular Carcinoma in Dual-Phase Magnetic Resonance Images

**DOI:** 10.1155/2024/9267554

**Published:** 2024-02-28

**Authors:** Yufeng Ye, Naiwen Zhang, Dasheng Wu, Bingsheng Huang, Xun Cai, Xiaolei Ruan, Liangliang Chen, Kun Huang, Zi-Ping Li, Po-Man Wu, Jinzhao Jiang, Guo Dan, Zhenpeng Peng

**Affiliations:** ^1^The First Clinical College of Jinan University, Guangzhou, China; ^2^Department of Radiology, Panyu Central Hospital, Guangzhou, China; ^3^Medical AI Lab, School of Biomedical Engineering, Shenzhen University Medical School, Shenzhen University, Shenzhen, China; ^4^Shenzhen University Clinical Research Center for Neurological Diseases, Shenzhen, Guangdong, China; ^5^Jiuquan Satellite Launch Center, Lanzhou, Gansu, China; ^6^Department of Radiology, First Affiliated Hospital, Sun Yat-sen University, Guangzhou, China; ^7^Department of Radiology, Guizhou Provincial People's Hospital, Guiyang, China; ^8^Medical Physics and Research Department, Hong Kong Sanatorium & Hospital, Hong Kong, China; ^9^Department of Radiology, Shenzhen University General Hospital, Shenzhen, China; ^10^School of Biomedical Engineering, Shenzhen University Medical School, Shenzhen University, Shenzhen, China

## Abstract

**Purpose:**

Segmentation of hepatocellular carcinoma (HCC) is crucial; however, manual segmentation is subjective and time-consuming. Accurate and automatic lesion contouring for HCC is desirable in clinical practice. In response to this need, our study introduced a segmentation approach for HCC combining deep convolutional neural networks (DCNNs) and radiologist intervention in magnetic resonance imaging (MRI). We sought to design a segmentation method with a deep learning method that automatically segments using manual location information for moderately experienced radiologists. In addition, we verified the viability of this method to assist radiologists in accurate and fast lesion segmentation.

**Method:**

In our study, we developed a semiautomatic approach for segmenting HCC using DCNN in conjunction with radiologist intervention in dual-phase gadolinium-ethoxybenzyl-diethylenetriamine penta-acetic acid- (Gd-EOB-DTPA-) enhanced MRI. We developed a DCNN and deep fusion network (DFN) trained on full-size images, namely, DCNN-F and DFN-F. Furthermore, DFN was applied to the image blocks containing tumor lesions that were roughly contoured by a radiologist with 10 years of experience in abdominal MRI, and this method was named DFN-R. Another radiologist with five years of experience (moderate experience) performed tumor lesion contouring for comparison with our proposed methods. The ground truth image was contoured by an experienced radiologist and reviewed by an independent experienced radiologist.

**Results:**

The mean DSC of DCNN-F, DFN-F, and DFN-R was 0.69 ± 0.20 (median, 0.72), 0.74 ± 0.21 (median, 0.77), and 0.83 ± 0.13 (median, 0.88), respectively. The mean DSC of the segmentation by the radiologist with moderate experience was 0.79 ± 0.11 (median, 0.83), which was lower than the performance of DFN-R.

**Conclusions:**

Deep learning using dual-phase MRI shows great potential for HCC lesion segmentation. The radiologist-aided semiautomated method (DFN-R) achieved improved performance compared to manual contouring by the radiologist with moderate experience, although the difference was not statistically significant.

## 1. Introduction

Magnetic resonance imaging (MRI) plays a crucial role in the clinical management of hepatocellular carcinoma (HCC). Segmentation of HCC on MRI helps clinicians obtain diagnostic insight on the disease, and treatment recommendations are usually made based on accurate volume measurement of the tumor. Radiologic tumor response assessment is also frequently performed according to tumor size evaluations [1]. Although manual segmentation of HCC is reliable, it is time-consuming and labor-intensive. In addition, strong subjectivity is observed, and accuracy varies depending on the experience of the radiologists [2].

HCC lesion contouring with computer-aided segmentation algorithms is reproducible and stable [3]. To date, a variety of HCC segmentation methods based on shallow handcrafted features have been proposed, most of which are problem-dependent [4–7] and thus limited in representing high-dimensional features of the lesions. Among these methods, machine learning methods [8, 9] have become increasingly popular. Foruzan and Chen [9] proposed a method based on a support vector machine (SVM) and achieved an average Dice similarity coefficient (DSC) score of 0.82. Deep learning (DL), a branch of machine learning, has been increasingly employed in medical image segmentation owing to its ability to extract highly informative and representative features. DL models are primarily composed of multiple processing layers to learn multiple levels of abstract features [10]. Deep convolutional neural networks (DCNNs), which are the most common DL networks, learn simple features (such as signal intensity, edges, and textures) as components of more complex features such as shapes, lesions, or organs, thereby leveraging the compositional nature of images [11]. Li et al. [12] developed a patch-wise convolutional neural network (CNN) for HCC segmentation and achieved an average DSC score of 0.80 with CT images. Li et al. [13] and Duc et al. [14] developed multitask deep learning and a CNN for HCC segmentation using CT images, which achieved average and median DSC scores of 0.74 and 0.81, respectively. These authors found that the DL method enabled accurate segmentation of HCC.

Christ et al. [15] proposed a cascaded fully convolutional neural network to perform HCC segmentation in diffusion-weighted MRI, reaching an average DSC of 0.69, which may not be sufficient for clinical use. This may be because Christ et al.'s HCC segmentation method [15] is based on single-phase MRI images, which may lack sufficient information for tumor lesion segmentation. The utilization of multiple phases could boost the accuracy of DL-based segmentation. Currently, few studies have explored the applications of DL on multiphase MRI images for tumor segmentation. Havaei et al. [16] attempted to incorporate multiple sequences (T1 weighted, T1WI; T2 weighted, T2WI; T1 contrast-enhanced; and fluid-attenuated inversion recovery) into a brain tumor segmentation task. With single-sequence MRI, the highest average DSC was 0.49, whereas using the four sequences helped boost the average DSC to 0.75. Regarding HCC detection on multisequence MRI images, Fabijańska et al. [17] achieved a detection accuracy of 0.98 using a CNN with optimized hyperparameter settings. Bousabarah et al. [18] used multiphasic T1-weighted images to segment the HCC with DCNN. This yielded an average DSC score of 0.64 and 0.68 in the validation and test cohort, respectively. Zheng et al. [19] proposed a four-dimensional deep learning model based on three-dimensional (3D) convolution and convolutional long short-term memory for HCC lesion segmentation using multiphase dynamic contrast-enhanced (DCE) MRI images. This method yielded an average DSC score of 0.83 highlighting the potential of multiphase MRI in HCC segmentation.

Gadolinium-ethoxybenzyl-diethylenetriamine penta-acetic acid (Gd-EOB-DTPA), a new hepatobiliary-specific MRI contrast agent [20], has been widely used in HCC diagnosis and presurgical evaluation [21]. In the hepatobiliary phase (HBP) of Gd-EOB-DTPA-enhanced MRI, HCC usually presents a low signal intensity compared with the surrounding liver tissue. The difference in signal between tumor tissues and the surrounding liver parenchyma is more prominent. This highlights the tumor boundaries allowing delineation and providing valuable information for HCC lesion segmentation. This study is aimed at investigating the efficacy of a dual-phase DL model on Gd-EOB-DTPA-enhanced MRI images for HCC segmentation. Although several automated segmentation methods have been proposed, no method has achieved stable and high performance without inspection by clinicians [6]. Previous studies [5, 6, 22, 23] have demonstrated that clinicians remain indispensable for correcting false segmentation after automatic segmentation. Thus, we aimed to design a segmentation approach for moderately experienced radiologists using a deep learning method that automatically segments according to manual location information. In addition, we verified whether this method is a viable solution for assisting radiologists in accurate and fast lesion segmentation.

## 2. Materials and Methods

### 2.1. Patients and MRI Acquisition

MRI images of patients with newly diagnosed HCC were retrospectively collected from January 2012 to June 2015. The ethics committee performed the ethical review, approved this study, and waived the requirement for informed written consent from the patients. This dataset included 51 patients (46 men, five women; age range, 29–84 years) who underwent contrast-enhanced MRI. MRI scans were conducted on a 3.0 T scanner (Magnetom Trio; Siemens Healthineers, Erlangen, Germany) using a body coil in the supine position. The Gd-EOB-DTPA-enhanced MRI scanning protocol included the following sequences: T1WI, T2WI, fat-suppressed T1WI pre-enhanced, dynamic-enhanced fat-suppressed volumetric interpolated breath-hold T1WI examination (including arterial phase, portal venous phase, and delayed phase), and hepatobiliary phase scanning (20 min after injection). In this study, two Gd-EOB-DTPA contrast-enhanced T1-weighted series including the hepatobiliary phase (HBP) and portal venous phase (PVP) were utilized to construct deep learning models. HBP-MRI scanning was performed using the following parameters: TR = 4.37 ms; TE = 1.42 ms; field of view, 328 × 350 mm^2^; and flip angle, 13°. PVP-MRI scanning was performed using the following parameters: TR = 3.92 ms, TE = 1.39 ms, field of view = 328 × 350 mm^2^, and flip angle=9°. The reconstructed images had a matrix size of 512 × 512 and 320 × 320, respectively, and a slice thickness of 5 mm and 2 mm, respectively.

### 2.2. Experimental Design

**Figure 1:**
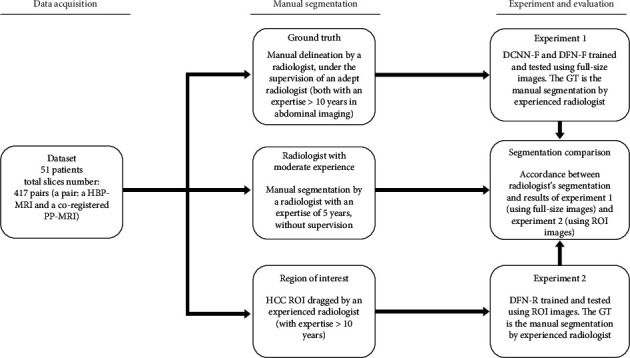
Flowchart of the proposed experimental framework.

A flowchart of the proposed experimental framework is shown in Figure 1. We performed two experiments using our dataset (HBP-MRI and PVP-MRI images of 51 patients).

**Figure 2:**
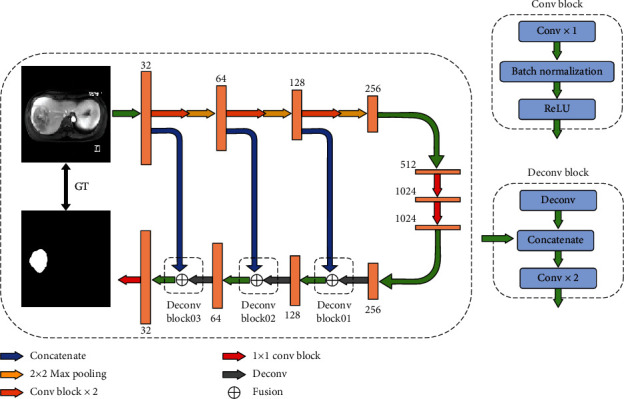
Architecture of the proposed deep convolutional neural networks (DCNNs). The proposed network includes two phases, contracting path (the upper and middle part of the network) and the expansive path (the lower part of the network). The contracting path is composed of six convolutional (conv) blocks (a block consists of the repeated application of a 3 × 3 conv, batch normalization, and ReLU), three max pooling layers, and two 1 × 1 conv layers. Every two conv blocks are followed by a 2 × 2 max pooling layer with stride two. The expansive path is composed of three upsampling blocks (deconv block). Each Deconv Block consists of an upsampling of the feature map followed by a 2 × 2 deconv, a concatenation with the correspondingly cropped feature map from the contracting path, and two 3 × 3 convs.

**Figure 3:**
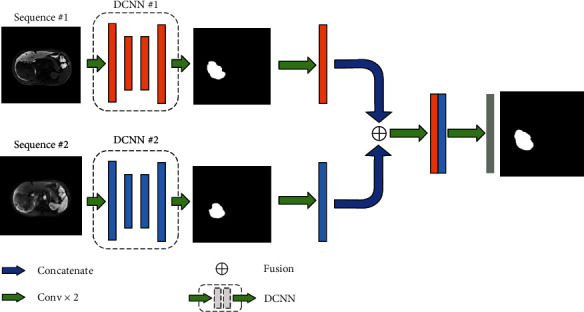
Architecture of the proposed deep fusion network (DFN). DFN includes 2 components: subdeep convolutional neural networks (DCNN) and fusion blocks. The input data initially go through different sub-DCNNs, and a fusion block gathers their segmentation score maps and integrates them to reconstruct the final delineation outcome.

In experiment 1, we evaluated the performance of the proposed DCNN (Figure 2) trained using full-size single-phase MRI and the deep fusion network (DFN, Figure 3) with full-size dual-phase MRI. These were named DCNN-F and DFN-F, respectively. The detailed architecture of the DCNN and DFN can be found in Section 2.4. HBP-MRI was chosen as the input sequence of DCNN-F because of its excellence in differentiating HCC from its background. Additionally, HBP-MRI and PVP-MRI were used as the input for DFN-F. PVP-MRI may provide a unique characterization of HCC, in contrast to HBP-MRI.

In experiment 2, to improve segmentation accuracy, radiologist intervention was introduced by predefining tumor lesion regions of interest (ROIs) in the MRI images (details of the predefined procedure are illustrated below). Additionally, the automatic segmentation was trained and tested only on the ROI images using the same DFN as in experiment 1 (for distinguishing from experiment 1, we named it the DFN-R model). HBP-MRI and PVP-MRI were also used as input images.

### 2.3. Manual Segmentation and Predefined ROI Drawing

The ground truth (GT) of the tumor was contoured carefully by a board-certified abdominal radiologist and reviewed by an independent radiologist (both with >10 years of expertise in abdominal imaging). Tumor lesion delineation was also performed independently by a radiologist with an expertise of five years (moderate experience) in abdominal imaging. This was performed without an experienced radiologist's supervision and was used only for efficacy comparisons with our proposed deep learning models.

For predefined ROIs of HCC, a radiologist with >10 years of experience was invited to review the MRI scans and roughly locate the tumor. This was done by dragging three rectangles on the *x*‐*y*, *x*‐*z*, and *y*‐*z* planes. A 3D tumor ROI was then determined using these rectangles. All ROIs were strictly examined to ensure that the target HCCs were completely included.

GT contouring, segmentation by a radiologist with moderate experience, and predefined ROI drawing were all performed in HBP-MRI images using ITK-SNAP (http://www.itksnap.org) [24].

### 2.4. Deep Learning-Based Segmentation

#### 2.4.1. Network Architectures

Two DL models, DCNN and DFN, were used in this study, for which the network architecture diagrams can be found in Figures 2 and 3.

Our proposed DCNN architecture was based on the fully convolutional network [25] and U-Net [26]. These tools have shown promising performance for medical image segmentation problems. As shown in Figure 2, in our DCNN architecture, we modified the number of pooling layers and added a batch normalization layer before the rectified linear unit layer to avoid an overfitting or vanishing gradient. Our DCNN model uses a Dice loss function to measure the difference between true segmentation and its output.

We adapted a model-based fusion scheme to build the DFN (Figure 3). With DFN, the images of both PVP-MRI and HBP-MRI went through the DCNN pipelines, and the initial segmentation score map of each phase was returned. These initial segmentation score maps were then input into the fusion block, where they were further integrated into the final segmentation score map. The DFN architecture can be described as follows:

(1) $$\begin{array}{c} H\left(P,W\right)=f\left[\overset{N}{\underset{i=1}{\sum }}o\left(P_{i}\right),W_{i}\right], \end{array}$$

where *P*_*i*_ and *o*(*P*_*i*_) represent the input and output of the *i*-th sub-DCNN, respectively, *W*_*i*_ denotes the weights of the *i*-th DCNN, and *f* is the mapping function of the DFN. Multiple DCNN weights are fine-tuned in training. Therefore, the actual loss function of the DFN is

(2) $$\begin{array}{c} L\left(G,P;\alpha \right)=\overset{N}{\underset{i=1}{\sum }}L_{\text{subne}{\text{t}}_{i}}\left(G,P_{i}\right)\times \alpha_{i}+L_{\text{DFN}}\left(G,P_{\text{out}}\right), \end{array}$$

where *L*_subnet_*i*__(*G*, *P*_*i*_) is the loss of the *i*-th DCNN, *α*_*i*_ represents the loss weights of the *i-*th sub-DCNN, *P*_out_ is the final output of the DFN, and *L*_DFN_(*G*, *P*_out_) is the measurement of the loss between the outputs of the DFN and GT. *N* defines the number of sub-DCNNs. We set *N* = 2 because two MRI phases (HBP-MRI and PVP-MRI) were used as inputs in our study.

#### 2.4.2. Image Preprocessing

To better utilize the information from both phases, each PVP-MRI image was registered to the corresponding HBP-MRI image via a B-spline nonrigid registration algorithm implemented using SimpleITK [27, 28]. A larger input matrix size requires more powerful graphic processing units and training time. To reduce the computation time and unify the inputs for the DCNN and DFN, we used two-dimensional (2D) images as the input of the networks and resampled them to 256 × 256.

#### 2.4.3. Network Training and Testing

For network training, image slices with tumors were selected, and we collected 417 pairs of images (each pair contained an HBP-MRI slice and its coregistered PVP-MRI slice). DL requires a large amount of data to complete the learning process. One solution to this problem is data augmentation. We augmented the training dataset to nearly 52,000 samples (26,000 pairs of images). Additionally, data augmentation methods included image rotation in the range of −20° to 20°, image scaling in the range of 0.9–1.1, contrast adjustment in the range of -5 to 5, and adding noise and horizontal mirroring. All data augmentations were implemented by using Pillow (https://pypi.org/project/Pillow/).

The training of the DCNN contained three main steps. Firstly, the feature extraction pathway of the DCNN, which was integral for extracting key features from the input data (the contracting path in Figure 2), was trained using nonaugmented training data. Subsequently, the same nonaugmented data was employed to train the entire network, a process aimed at refining the network's parameters and ensuring its effective learning from the original data. The final step involved utilizing data augmentation techniques to create new training samples, thereby bolstering the model's generalization capability and enhancing its performance in practical scenarios. Fine-tuning the DCNN with these augmented data samples was crucial for adapting the model to novel, unseen data, consequently elevating its robustness and accuracy in real-world applications. The training set contained 50 patients using the leave-one-out cross-validation (LOOCV) strategy. GT was contoured by an experienced radiologist and reviewed by an independent experienced radiologist.

When the DFN integrates multiple DCNN results, the optimal weights for these sub-DCNNs may not be optimal for the entire DFN. Therefore, the training dataset is randomly split into two equal subsets. The two sub-DCNNs were first trained with one subset using the same training strategy as above. Another subset was used to train the entire DFN after the weights of the two sub-DCNNs were established. Fine-tuning the fusion block after setting the initial weights of the sub-DCNNs may refine the results. The following parameters were used for training: Adam optimizer and stochastic gradient descent for DCNN and DFN, respectively. The base learning rate for DCNN and DFN was 1 × 10^−4^ and 1 × 10^−7^, respectively. The batch size was eight.

A leave-one-out cross-validation strategy was applied from the beginning. Using this strategy, we randomly selected one patient as the testing set each time and trained the network using the remaining patients. For testing, images of the patient used as the testing dataset were input into the trained model to perform the segmentation task. To evaluate the performance of the models, we adopted the DSC score, recall, and precision as the performance metrics. True positive (TP) signifies a correctly identified tumor area, false positive (FP) denotes an area where normal tissue is wrongly identified as a tumor, and false negative (FN) denotes a tumor area that is incorrectly identified as normal tissue. The DSC score, precision, and recall are defined as follows:

(3) $$\begin{array}{c} \text{DSC}=\frac{2\text{TP}}{\text{FP}+2\text{TP}+\text{FN}},\\ \text{Precision}=\frac{\text{TP}}{\text{TP}+\text{FP}},\\ \text{Recall}=\frac{\text{TP}}{\text{TP}+\text{FN}}. \end{array}$$

Training and testing were performed on a workstation with a Linux system (Ubuntu 14.04; Canonnical, London, England). The workstation used a Processor E5-2650 v3 CPU and NVIDIA GeForce 1080TI GPU. The DL framework Keras [29] and TensorFlow [30] back-end were used for implementing our network architectures.

### 2.5. Statistical Analysis

Statistical comparisons between the DSC scores of our models and the segmentation by a radiologist with moderate experience were performed using a paired *t*-test. *p* < 0.05 indicates that there was a statistically significant difference in the DSC score between the segmentation results of our models and that of the radiologist (moderate experience). All statistical analyses were performed using SPSS (version 20.0; SPSS Inc., Chicago, ILL, USA).

## 3. Results

A summary of the segmentation results by our proposed methods and by a radiologist with moderate experience is presented in Table 1.

**Figure 4:**
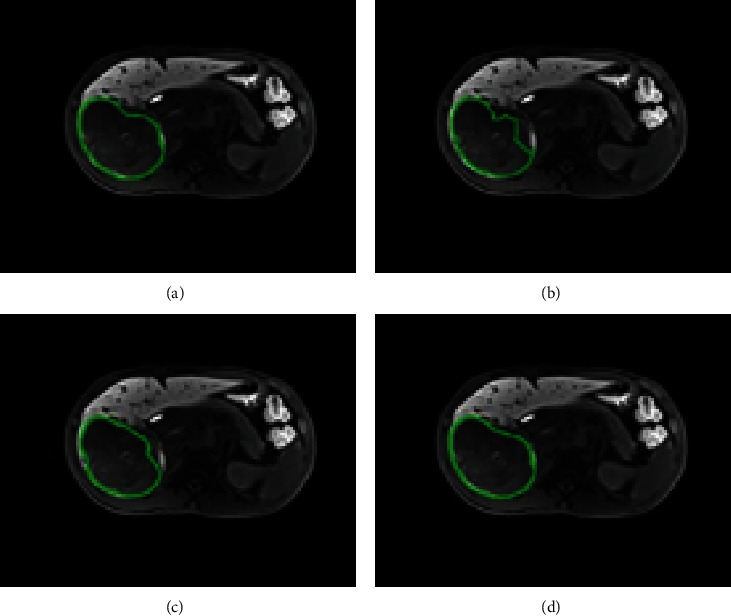
A typical example of hepatocellular carcinoma (HCC) segmentation with high accuracy. The Dice similarity coefficient (DSC) was 0.86 and 0.92 for deep fusion network- (DFN-) F and DFN-R, respectively. All contouring results are presented on hepatobiliary phase- (HBP-) magnetic resonance imaging (MRI). (a) Ground truth (GT). (b) Segmentation result of deep convolutional neural network- (DCNN-) F. (c) Segmentation result of DFN-F. (d) Segmentation result of DFN-R.

With respect to DCNN-F and DFN-F in experiment 1, the mean DSC of 51 patients was 0.69 (0.01–0.90; median, 0.72) and 0.74 (0.08–0.96; median, 0.77), respectively. The mean precision was 0.70 (0.01–0.91; median, 0.78) and 0.70 (0.01–0.96; median, 0.72), respectively. The mean recall was 0.72 (0.01–0.89; median, 0.75) and 0.78 (0.01–0.98; median, 0.91), respectively. For DFN-R in experiment 2, the mean DSC was 0.83 (0.42–0.96; median, 0.88), the mean precision was 0.81 (0.21–0.99; median, 0.87), and the mean recall was 0.88 (0.48–0.99; median, 0.91). An example of DFN-F segmentation with high accuracy is presented in Figure 4, in which the DSC of DFN-F and DFN-R was 0.86 and 0.92, respectively.

**Figure 5:**
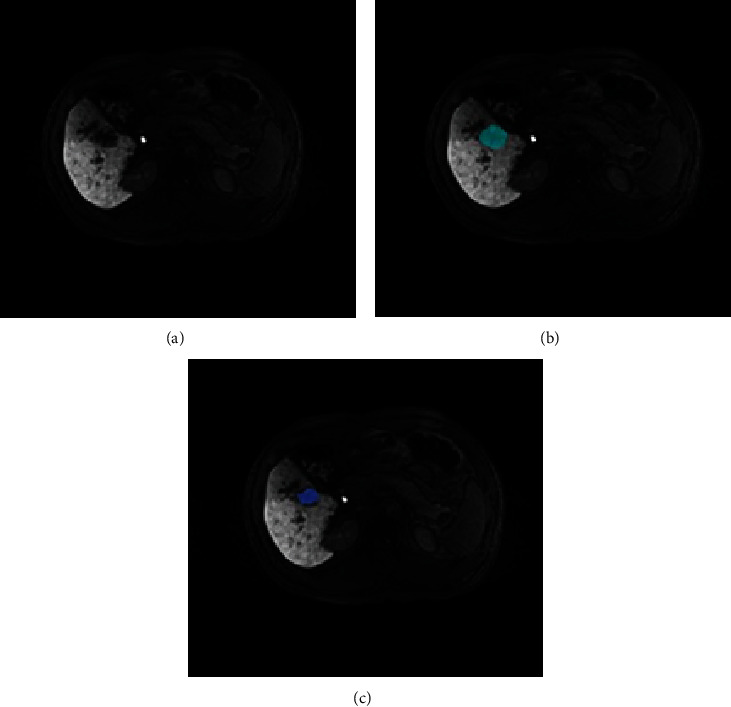
An example of discrepancy between ground truth (GT) and segmentation by the radiologist with moderate experience. The original hepatobiliary phase- (HBP-) magnetic resonance imaging (MRI) image was presented (a). (b) GT and (c) segmentation by the radiologist with moderate experience were presented in indigo and blue, respectively. The radiologist with moderate experience tended to contour a smaller border when the hepatocellular carcinoma (HCC) lesion had unclear margins.

The average DSC of the segmentation results by the radiologist with moderate experience was 0.79 (0.45–0.96; median, 0.83), as compared to the GT contoured by the two radiologists with >10 years of experience. The results obtained by the radiologist with moderate experience were consistent with the GT of the tumor, with a clear border and homogeneous intensity. However, some segmentation results may differ from those of GT, especially when the tumor lesion is small or heterogeneous (Figure 5).

Compared to segmentation by the radiologist with moderate experience, the mean DSC by DCNN-F was significantly lower (*p* = 0.03), while the results of DFN-F (*p* = 0.26) and DFN-R (*p* = 0.69) did not differ significantly. However, the average DSC and median DSC of DFN-R were both higher than those of the radiologist with moderate experience (mean, 0.83 versus 0.79; median, 0.88 versus 0.83).

Approximately 1 min was required to determine a 3D tumor ROI rectangle, 10 min to train the network for a fold in LOOCV, and 1.2 s to segment a lesion using 50 slices of an MRI image. Therefore, the total time required by the DFN-R model was approximately 11 min. For a radiologist with moderate experience, approximately 15 min was required to delineate an HCC tumor using the HBP-MRI images. Deep learning-based segmentation was faster than manual contouring by radiologists.

To evaluate the performance of our models, we compared our results to those of previous studies [8, 9, 12, 13, 15]. Reproducing their experimental results was challenging. This may be due to the specific parameter settings and database. Therefore, we directly compared our results with those of these studies in terms of DSC. Although they may not be reasonably comparable, these assessments provide insight into how our method outperformed similar studies. The results of the previous studies on HCC segmentation are presented in Table 2. Among these methods, the proposed DFN-R model (average DSC, 0.83) achieved the highest segmentation accuracy.

## 4. Discussion

Our experimental results show that HCC can be efficiently segmented using the proposed DFN model. Manual segmentation by experienced radiologists is usually considered accurate and stable. However, the mean DSC of segmentation by the radiologist with moderate experience in this study was 0.79 (Table 1), and the proposed DFN-R model achieved slightly improved performance compared to that radiologist.

**Figure 6:**
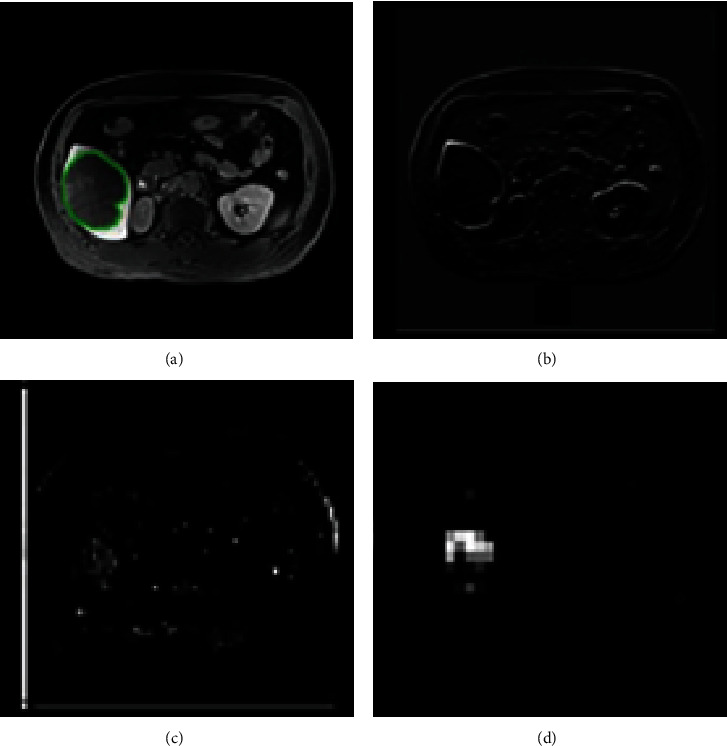
The heatmaps of convolutional layers at different stages of (b–d) deep convolutional neural networks (DCNNs) of an (a) input image. The ground truth (GT) of hepatocellular carcinoma (HCC) margins was presented on the patient's hepatobiliary phase- (HBP-) magnetic resonance imaging (MRI) image. The border of the abdomen and other organs can be overserved at the shallow layer ((b) conv2_1). As the network progressed deeper, less details were kept, but the border of the abdomen was discernible ((c) conv4_1). Finally, the HCC retained a high response, while other regions were excluded ((d) deconv3_1).

**Figure 7:**
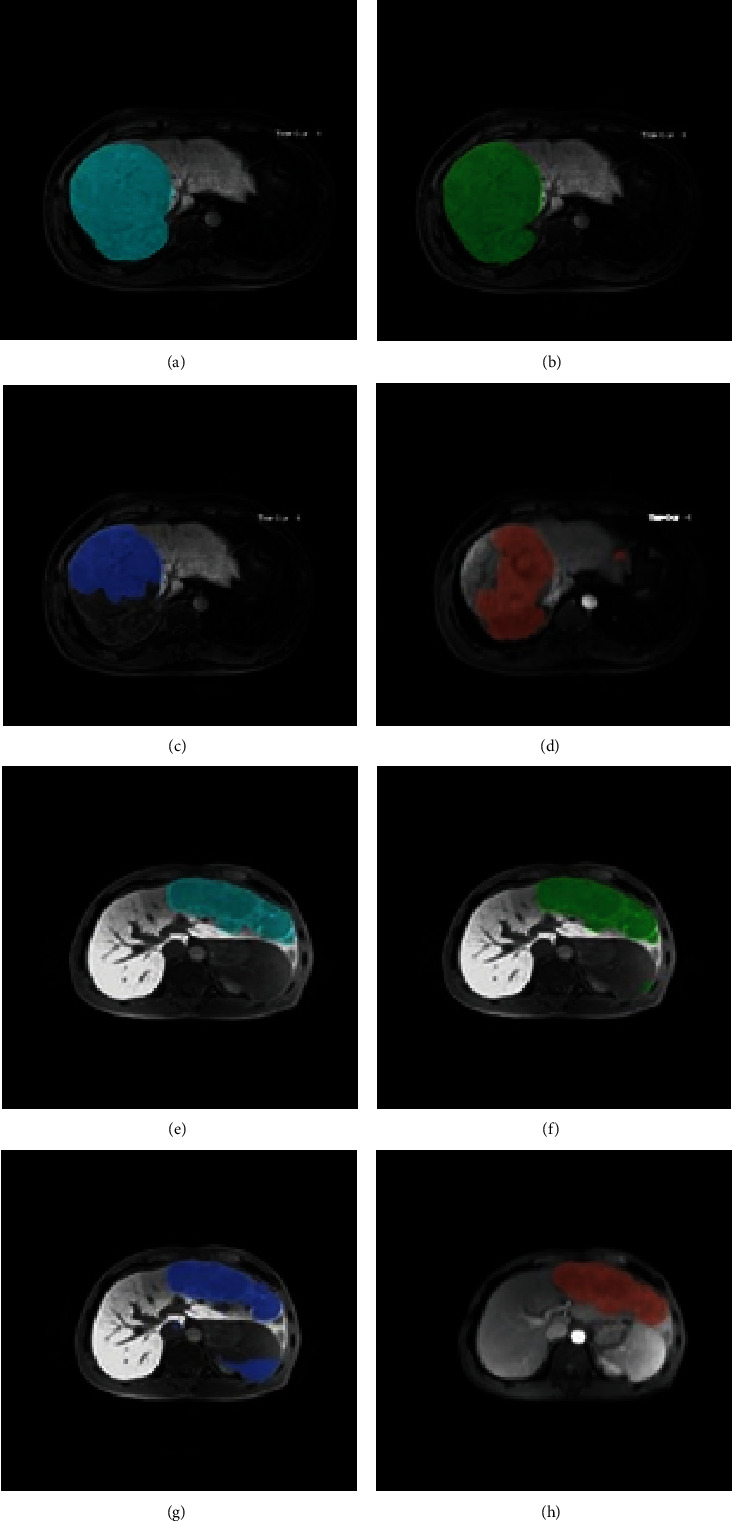
Two examples of the segmentation results by deep fusion network- (DFN-) F. (a, e) Ground truth (GT) (first column), (b, f) segmentation results by DFN-F (second column), (c, g) subdeep convolutional neural networks (DCNNs) (in hepatobiliary phase- (HBP-) magnetic resonance imaging (MRI), third column), and (d, h) sub-DCNN (portal venous phase- (PVP-) MRI, fourth column) are presented in indigo, green, blue, and light red, respectively. In the (a–d) first example, two (c, d) sub-DCNNs successfully recognized only part of the hepatocellular (HCC), while DFN-F successfully segmented the HCC lesions correctly. In the (e–h) second example, the sub-DCNN in (g) HBP-MRI misclassified normal tissues as tumor lesions, while DFN-F avoided false segmentation by taking the information of both (h) PVP-MRI and (g) HBP-MRI into consideration.

Our DCNN architecture was able to detect tumors by extracting deep representations of HCC, as shown in the heatmaps (Figure 6) of convolutional layers at different stages of the DCNN. However, it has disadvantages, as shown in Figures 7(a)–7(d). Only part of the tumor region was recognized by the sub-DCNN with HBP-MRI, while the unidentified part of the tumor was successfully segmented by the other sub-DCNN with PVP-MRI. Indeed, DCNN architecture attained a DSC of 0.69, comparable to existing HCC segmentation methods utilizing deep learning [15]. However, this level of accuracy, while in line with current standards, remains suboptimal. This rather unsatisfactory accuracy may be because DCNN only utilized one sequence: this method cannot contour the tumor's boundary precisely or prevent wide-range false negative predictions owing to insufficient information acquired from a single sequence. In contrast, our DFN-F model, by harnessing data from dual-phase MRI, demonstrated superior performance in HCC segmentation, as evidenced by the results with dual-phase MRI images (Table 1).

DCNN cannot contour the tumor's boundary precisely or prevent wide-range false negative predictions owing to insufficient information acquired from a single sequence. Our DFN-F model successfully utilized information from dual-phase MRI and showed promising results for HCC segmentation with dual-phase MRI images (Table 1). HBP-MRI and PVP-MRI provided complementary information, resulting in improved contouring accuracy and fewer false negative predictions. In HBP-MRI, certain organs can present a low intensity similar to HCC, leading to the misclassification of normal regions as cancerous. PVP-MRI helps eliminate this error by providing normal intensity contrast (Figures 7(e)–7(h)). This method considers both segmentation results and refines the final contouring outcome with a more accurate border.

**Figure 8:**
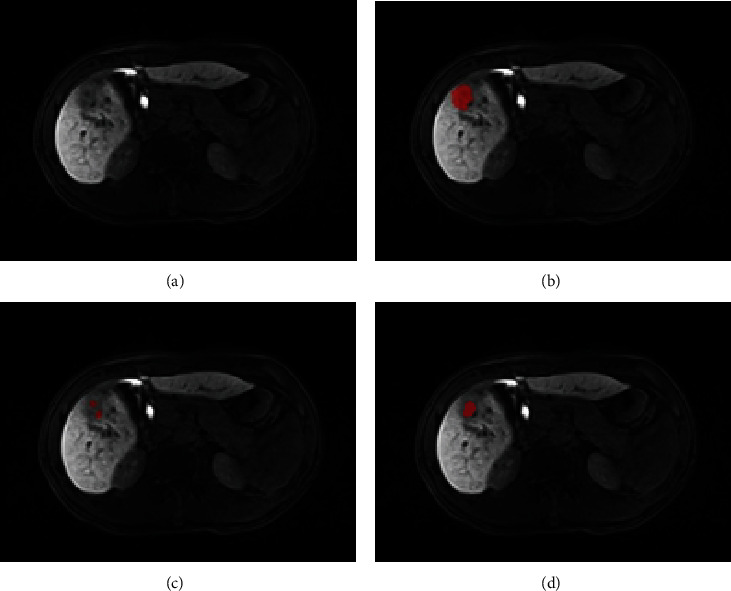
The example of segmentation by deep fusion network- (DFN-) F and DFN-R in which the Dice similarity coefficient (DSC) value was 0.08 and 0.42, respectively. (a) Original image, (b) ground truth GT, (c) segmentation by DFN-F, and (d) segmentation by DFN-R.

**Figure 9:**
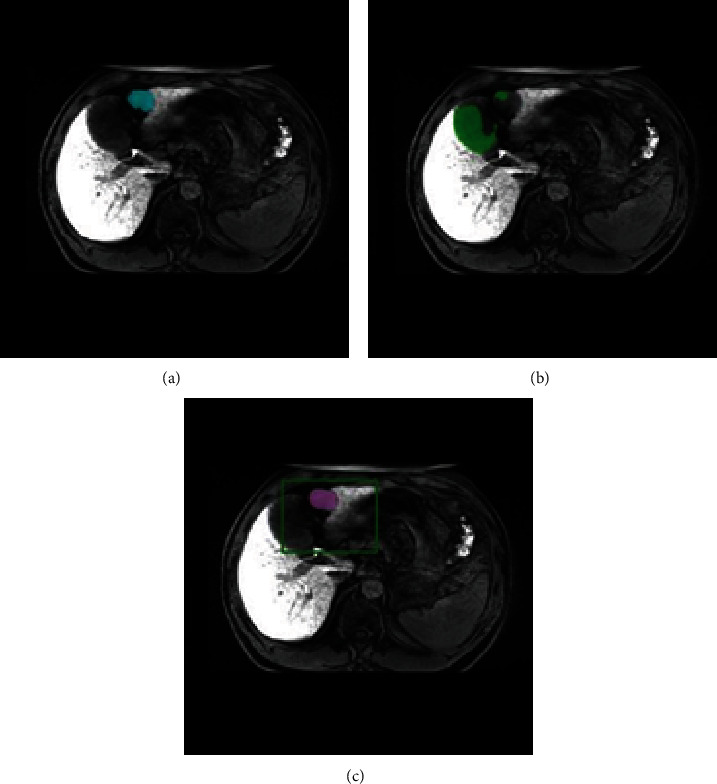
An example showing how deep fusion network- (DFN-) R helps improve segmentation accuracy. (a) Ground tumor (GT) and segmentation results by (b) DFN-F and (c) DFN-R on hepatobiliary phase- (HBP-) magnetic resonance imaging (MRI) are presented in indigo, green, and light purple, respectively. The intervention by the radiologist (green rectangular) differentiated the hepatocellular carcinoma (HCC) lesion from its surrounding tissue with similar intensity which may be misclassified as tumor lesions by DFN-F. Therefore, by using DFN-R, the target area is strictly restricted, and the lesion can be correctly distinguished and segmented.

The lowest DSC values for DCNN-F and DFN-F were equal to 0.01 and 0.08, respectively. This indicates a limitation of DFN-F in the segmentation tasks of certain tumors. After careful examination of these lesions (Figure 8), we observed that they shared an unclear HCC boundary, which may have been caused by complications such as cirrhosis. These complications often affect the absorption of contrast agents (Gd-EOB-DTPA) by normal cells around the lesion, resulting in inconspicuous contrast between the lesion and its surrounding normal tissue in Gd-EOB-DTPA-enhanced scanning. In these situations, the DL model is likely to misclassify HCC tissues from normal tissues. A potential mitigation to this problem is radiologist-aided intervention, in which a radiologist previews the patient's image and draws an ROI to define the tumor lesion region for DFN input. Using this intervention, in the experiment with DFN-R, we achieved a mean DSC of 0.83, which was significantly improved compared to that of DFN-F (0.83 versus 0.74). The performance of the DFN-R was comparable to the findings (DSC = 0.83) reported by Zheng et al. [19], who employed a completely automatic segmentation approach. Nonetheless, the DCE-MRI employed in their study demands an intricate analysis of multiple image phases to derive segmentation results, imposing stringent requirements on image acquisition. In contrast, Gd-EOB-DTPA contrast-enhanced MRI offers superior advantages over DCE-MRI in the diagnosis, treatment decision-making, efficacy assessment, and prognosis prediction for focal liver lesions. In clinical practice, Gd-EOB-DTPA contrast-enhanced MRI is the preferred choice. Intervention by the radiologist helped exclude irrelevant information and isolate the target region from the background (Figure 9). Moreover, the predefined ROI served as prior knowledge of the lesion location for DFN.

Segmentation by a radiologist with moderate experience showed good agreement with GT on lesions with clear borders and homogeneous density. However, segmentation by a radiologist with moderate experience showed a discrepancy with the GT when the tumor was small or had heterogeneous intensity (Figure 5). Such variability in segmentation results may indicate that manual contouring of the tumor is highly affected by the radiologists' experience. Therefore, a method that can produce stable and accurate HCC segmentation outcomes is desirable. In comparison with the segmentation results by the radiologist with moderate experience, DFN-R reached higher performance (mean DSC, 0.83 ± 0.13 versus 0.79 ± 0.11). In addition, the DFN-R provides a more stable and faster method for HCC segmentation. Therefore, this semiautomatic segmentation strategy may have potential applications in future clinical practice.

The present study has several limitations. First, our proposed DFN-R included an intervention from an experienced radiologist, which made it a semiautomated method. Moreover, this approach was potentially influenced by the radiologist's subjective judgment. A fully automated DL-based method may be a more practical solution for clinical application [18]. Second, a 2D network structure was used in this study, and the 3D network structure may have a better performance [31]. However, 3D neural networks require significant computational resources [32, 33]. Future studies should focus on the utilization of 3D neural networks to explore how spatial information helps improve segmentation accuracy. Finally, our proposed method requires validation in future research using a larger sample recruited across different scanners or centers.

In summary, we proposed and verified a radiologist-aided dual-phase MRI segmentation framework based on DL for HCC delineation. Information from dual-phase MRI images can assist in segmenting HCC lesions. Our DFN model with radiologists' intervention (semiautomated method) yielded higher accuracy than a radiologist with moderate experience. This radiologist-aided method can be utilized in clinical practice. Finally, before clinical use, our proposed method requires validation in future research using a larger sample recruited across different scanners and centers.

## Figures and Tables

**Table 1:** Results of DCNN-F, DFN-F, DFN-R, and the segmentation by the radiologist with moderate experience. Comparisons of performance were performed by using paired *t*-test.

Model	DSC			Precision			Recall			*p* value^∗^
	Mean ± std	Median	Range	Mean ± std	Median	Range	Mean ± std	Median	Range	
DCNN-F	0.69 ± 0.20	0.72	0.01~0.90	0.70 ± 0.24	0.78	0.01~0.91	0.72 ± 0.19	0.75	0.01~0.89	0.03
DFN-F	0.74 ± 0.21	0.77	0.08~0.96	0.70 ± 0.22	0.72	0.01~0.96	0.78 ± 0.29	0.91	0.01~0.98	0.26
DFN-R	0.83 ± 0.13	0.88	0.42~0.96	0.81 ± 0.16	0.87	0.21~0.99	0.88 ± 0.11	0.91	0.48~0.99	0.69
Radiologist with moderate experience	0.79 ± 0.11	0.83	0.45~0.96	0.86 ± 0.16	0.94	0.34~0.99	0.79 ± 0.12	0.75	0.47~0.99	—

^∗^A *p* value < 0.05 indicates that DSC results of one model are significantly different from those by the radiologist with moderate experience. Notes: DCNN: deep convolutional neural networks; DFN: deep fusion network; DSC: Dice similarity coefficient; std: standard deviation.

**Table 2:** Comparisons of segmentation performance between DFN-R and previous studies.

Studies	Algorithm	Images	Average DSC	Patient number	Journal
Linguraru [8]	SVM	CT	0.74	101	IEEE TMI, 2012
Foruzan [9]	SVM	CT	0.82	35	IJCARS, 2016
Li [12]	CNN	CT	0.80	30	JCC, 2017
Li [13]	CNN	CT	0.74	248	J PERS MED, 2022
Christ [15]	CFCNs	DW-MRI	0.69	31	MICCAI, 2016
Fabijańska [17]	U-Net	DCE-MRI	0.48	9	ICCVG, 2018
Khaled [18]	DCNN	T1-weighted MRI	0.68	174	ABDOM RADIOL, 2020
Zheng [19]	CNN	DCE-MRI	0.83	190	IEEE TMI, 2020
Current study	DFN-R	HBP-MRI and PVP-MRI	0.83	51	**—**

Notes: MRI: magnetic resonance imaging; DCNN: deep convolutional neural networks; DFN: deep fusion network; SVM: support vector machine; CNN: convolutional neural networks; CFCNs: cascaded fully convolutional neural networks; DW-MRI: diffusion-weighted MRI; DCE-MRI: dynamic contrast-enhanced MRI.

## Data Availability

All data used during the study are available from the corresponding authors by request after being published.

## References

[1] Bruix J., Sherman M. (2005). Management of hepatocellular carcinoma. *Hepatology*.

[2] Bellon E., Feron M., Frederik M. (1997). Evaluation of manual vs semi-automated delineation of liver lesions on CT images. *European Radiology*.

[3] Chai H., Guo Y., Wang Y., Zhou G. (2017). Automatic computer aided analysis algorithms and system for adrenal tumors on CT images. *Technology and Health Care*.

[4] Hoogi A., Beaulieu C. F., Cunha G. M. (2017). Adaptive local window for level set segmentation of CT and MRI liver lesions. *Medical Image Analysis*.

[5] Tacher V., Lin M. D., Chao M. (2013). Semiautomatic volumetric tumor segmentation for hepatocellular carcinoma: comparison between C-arm cone beam computed tomography and MRI. *Academic Radiology*.

[6] Wang Z., Chapiro J., Schernthaner R. (2015). Multimodality 3d tumor segmentation in Hcc patients treated with Tace. *Academic Radiology*.

[7] Le T.-N., Huynh H. T. (2016). Liver tumor segmentation from MR images using 3D fast marching algorithm and single hidden layer feedforward neural network. *BioMed Research International*.

[8] Linguraru M. G., Richbourg W. J., Liu J. (2012). Tumor burden analysis on computed tomography by automated liver and tumor segmentation. *IEEE transactions on medical imaging*.

[9] Foruzan A. H., Chen Y.-W. (2016). Improved segmentation of low-contrast lesions using sigmoid edge model. *International Journal of Computer Assisted Radiology and Surgery*.

[10] LeCun Y., Bengio Y., Hinton G. (2015). Deep learning. *Nature*.

[11] Chartrand G., Cheng P. M., Vorontsov E. (2017). Deep learning: a primer for radiologists. *Radiographics*.

[12] Li W., Jia F., Qingmao H. (2015). Automatic segmentation of liver tumor in CT images with deep convolutional neural networks. *Journal of Computer and Communications*.

[13] Li Y., Xu Z., An C., Chen H., Li X. (2022). Multi-task deep learning approach for simultaneous objective response prediction and tumor segmentation in HCC patients with transarterial chemoembolization. *Journal of Personalized Medicine*.

[14] Duc V. T., Chien P. C., Le Minh T. (2022). Deep learning model with convolutional neural network for detecting and segmenting hepatocellular carcinoma in CT: a preliminary study. *Cureus*.

[15] Christ P. F., Elshaer M. E. A., Ettlinger F., Ourselin S., Joskowicz L., Sabuncu M., Unal G., Wells W. Automatic Liver and Lesion Segmentation in CT Using Cascaded Fully Convolutional Neural Networks and 3D Conditional Random Fields. *Medical Image Computing and Computer-Assisted Intervention – MICCAI 2016. MICCAI 2016*.

[16] Havaei M., Guizard N., Chapados N., Bengio Y., Ourselin S., Joskowicz L., Sabuncu M., Unal G., Wells W. (2016). HeMIS: Hetero-Modal Image Segmentation. *Medical Image Computing and Computer-Assisted Intervention – MICCAI 2016. MICCAI 2016*.

[17] Fabijańska A., Vacavant A., Lebre M.-A., Chmielewski L., Kozera R., Orłowski A., Wojciechowski K., Bruckstein A., Petkov N. U-CatcHCC: An Accurate HCC Detector in Hepatic DCE-MRI Sequences Based on an U-Net Framework. *Computer Vision and Graphics. ICCVG 2018*.

[18] Bousabarah K., Letzen B., Tefera J. (2021). Automated detection and delineation of hepatocellular carcinoma on multiphasic contrast-enhanced MRI using deep learning. *Abdominal Radiology*.

[19] Zheng R., Wang Q., Lv S. (2022). Automatic liver tumor segmentation on dynamic contrast enhanced MRI using 4D information: deep learning model based on 3D convolution and convolutional LSTM. *IEEE Transactions on Medical Imaging*.

[20] Beers V., Bernard E., Pastor C. M., Hussain H. K. (2012). Primovist, Eovist: what to expect?. *Journal of Hepatology*.

[21] Saito K., Kotake F., Ito N. (2005). Gd-EOB-DTPA enhanced MRI for hepatocellular carcinoma: quantitative evaluation of tumor enhancement in hepatobiliary phase. *Magnetic Resonance in Medical Sciences*.

[22] Meillan N., Bibault J.-E., Vautier J. (2018). Automatic intracranial segmentation: is the clinician still needed?. *Technology in Cancer Research & Treatment*.

[23] Lassen B. C., Jacobs C., Kuhnigk J. M., Van Ginneken B., Van Rikxoort E. M. (2015). Robust semi-automatic segmentation of pulmonary subsolid nodules in chest computed tomography scans. *Physics in Medicine & Biology*.

[24] Yushkevich P. A., Piven J., Hazlett H. C. (2006). User-guided 3D active contour segmentation of anatomical structures: significantly improved efficiency and reliability. *Neuroimage*.

[25] Long J., Shelhamer E., Darrell T. Fully convolutional networks for semantic segmentation.

[26] Ronneberger O., Fischer P., Brox T., Navab N., Hornegger J., Wells W., Frangi A. (2015). U-Net: Convolutional Networks for Biomedical Image Segmentation. *Medical Image Computing and Computer-Assisted Intervention – MICCAI 2015. MICCAI 2015*.

[27] Lowekamp B. C., Chen D. T., Ibáñez L., Blezek D. (2013). The design of SimpleITK. *Frontiers in Neuroinformatics*.

[28] Yaniv Z., Lowekamp B. C., Johnson H. J., Beare R. (2018). SimpleITK image-analysis notebooks: a collaborative environment for education and reproducible research. *Journal of Digital Imaging*.

[29] Keras C. F. (2015). GitHub repository. https://githubcom/keras-team/keras.

[30] Abadi M., Barham P., Chen J. {TensorFlow}: a system for {large-scale} machine learning.

[31] Ji S., Xu W., Yang M., Kai Y. (2013). 3D convolutional neural networks for human action recognition. *IEEE Transactions on Pattern Analysis and Machine Intelligence*.

[32] Roth H. R., Le Lu A. S., Cherry K. M., Golland P., Hata N., Barillot C., Hornegger J., Howe R. A New 2.5D Representation for Lymph Node Detection Using Random Sets of Deep Convolutional Neural Network Observations. *Medical Image Computing and Computer-Assisted Intervention – MICCAI 2014. MICCAI 2014*.

[33] Prasoon A., Petersen K., Igel C., Lauze F., Dam E., Nielsen M., Mori K., Sakuma I., Sato Y., Barillot C., Navab N. (2013). Deep Feature Learning for Knee Cartilage Segmentation Using a Triplanar Convolutional Neural Network. *Medical Image Computing and Computer-Assisted Intervention – MICCAI 2013. MICCAI 2013*.

